# Comparative analysis of Nafion-functionalized few-layered *vs.* multilayered Ti_3_C_2_T_*x*_ MXene/SnS_2_ nanoflowers for ammonia detection with enhanced selectivity

**DOI:** 10.1039/d6ra00396f

**Published:** 2026-03-03

**Authors:** Waqas Saeed, Ye Tian, Irshad Ahmad Mir, Baoji Miao, Amna Manzoor, Surjyakanta Rana, Xing Chen

**Affiliations:** a School of Mechanical and Electrical Engineering, Henan University of Technology Zhengzhou 450001 China yetian@haut.edu.cn; b Henan International Joint Laboratory of Nano-Photoelectric Magnetic Material, School of Materials Science and Engineering, Henan University of Technology Zhengzhou 450001 China mirirshad09@gmail.com; c Colloidal and Computational Lab, Department of Chemistry, Government College University Faisalabad Faisalabad 38000 Pakistan; d Centre for Functional and Surface Functionalized Glass, Alexander Dubček University of Trenčín 911 50 Trenčín Slovakia

## Abstract

Ammonia (NH_3_) detection at room temperature under varying humidity conditions is crucial for environmental monitoring, industrial safety, and healthcare. However, ammonia detection remains a challenge due to the lack of sensitivity and selectivity of current room temperature based sensors. In this context, Ti_3_C_2_T_*x*_ MXene and flower-like SnS_2_ composites were investigated for NH_3_ sensing. A comparative analysis of multilayered and few-layered nanohybrid composites demonstrated that the few-layered configuration displayed a markedly enhanced response to ammonia. Its larger surface area, higher electrical conductivity, and higher number of active sites contribute to its superior gas diffusion and charge transfer properties. The composite was further functionalized with Nafion (NF) to enhance sensitivity and selectivity under humid conditions. In an optimized NF-functionalized few-layered Ti_3_C_2_T_*x*_/SnS_2_ sensor, we achieved a 12.11 response ratio towards 100 ppm NH_3_, with a response time of 42 s and a recovery time of 156 s, as well as excellent selectivity towards interfering gases. Reliability tests conducted in humid environments confirmed the excellent operational stability and durability of the proposed sensor. These experimental results demonstrate that the developed few-layered Ti_3_C_2_T_*x*_/SnS_2_/NF nanohybrid composite provides a highly durable, sensitive, and selective platform for NH_3_ detection in practical applications at room temperature.

## Abstract 2

Ammonia (NH_3_) detection at room temperature under varying environmental conditions is crucial for environmental monitoring, industrial safety, and healthcare.

## Graphical abstract

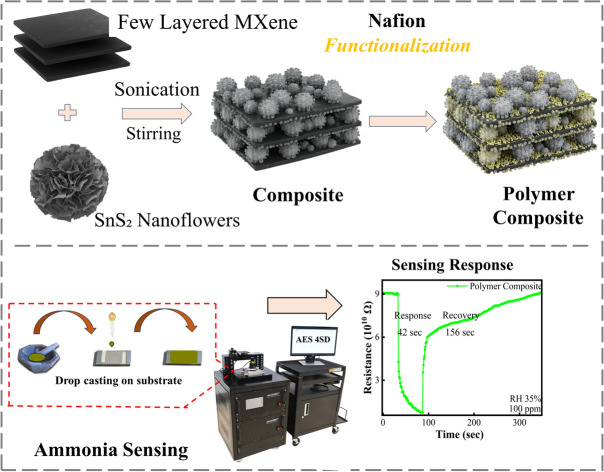

## Introduction

Our atmosphere contains a large amount of ammonia (NH_3_), which is primarily generated by three processes: bacterial nitrogen fixation, salt deposition, and combustion from vehicles and industries. It is a part of many raw materials, such as fertilizers, textiles, chemicals, and paper.^1^ However, NH_3_ is also a very harmful gas. Exposure to NH_3_ can result in headaches, vomiting, eye and lung irritation, and, at extremely high levels, even death. Any leak spreads swiftly over a large area due to its light and volatile nature.^2^ The hazards associated with NH_3_ exposure have been shown by health standards. A human nose can only detect it at levels above 25 ppm, but the Occupational Safety and Health Administration (OSHA) sets a safety limit of no more than 50 ppm at workplaces.^3^ Exposure to an ammonia concentration of approximately 100 ppm, even for a short duration, is believed to be hazardous to human health. Therefore, to address these serious issues associated with human health, it is of utmost importance to produce a low-cost, highly sensitive, and selective ammonia gas sensor.

Researchers have extensively focused on metal oxide-based NH_3_ gas sensors due to their low cost and simple fabrication method.^4,5^ These sensors have very limited practical applications due to their low selectivity and high operational temperature, *i.e.* 150–350 °C.^6,7^ To overcome these challenges, scientists have developed alternative materials, such as conducting polymers, transition metal dichalcogenides, transition metal carbides and their composites. In recent years, gas sensing research has focused a lot on MXenes. MXenes are two-dimensional (2D) transition metal carbides,^8,9^ with a general formula of M_*n*+1_X_*n*_T_*x*_ (*n* = 1, 2, or 3), where M is an early transition metal, X is carbon or nitrogen, and T_*x*_ is any surface termination group like –OH, –O, or –F.^10^ Researchers considered using multilayered Ti_3_C_2_T_*x*_, a single-transition-metal carbide MXene, in gas sensing applications because of its high conductivity, high functional group structure, and large specific surface area.^1,11,12^ In the stacked configuration, the nanosheets maintain good electrical conductivity due to the intrinsically metallic nature of Ti_3_C_2_T_*x*_ and the formation of continuous electron transport pathways. However, the close interlayer stacking restricts gas diffusion, limiting molecular accessibility and reducing the number of exposed active sites. Considering these structural constraints, adopting a very few-layered Ti_3_C_2_T_*x*_ form offers clear advantages. The few layers expose more surfaces and provide a larger space between the layers so that gas molecules can access them easily and charges can move faster. With its open structure, few-layered MXene promotes faster surface reactions, making it a more efficient and responsive material for surface-driven applications.^13^ MXenes are prone to oxidation when exposed to air or moisture and also suffer from low sensitivity, which limits their long-term stability. Therefore, they are often combined with other materials to form composites that enhance their structural integrity and environmental resistance.

Several transition metal dichalcogenides (TMDs) like WS_2_, MoS_2_, MoSe_2_, and SnS_2_ have received attention for ammonia gas detection in recent years because of their unique electronic properties resulting from their low-dimensional structure.^14–17^ In this category, two-dimensional transition metal dichalcogenides have been reported to form stable nanohybrids with other layered materials owing to their similar lattice structures and minimal lattice mismatch.^18^ Consequently, TMDs are now considered to be promising alternatives to traditional metal oxides used in ammonia gas sensing. Among transition metal dichalcogenides, SnS_2_ is characterized as a top-tier material because of its distinctive chemical and physical properties.^19^ With a narrow band gap of about 2.2 eV and a high electrical sensitivity to gas molecules, it is highly suitable for sensing ammonia at room temperature.^20^ However, factors such as humidity sensitivity and slow response or recovery still limit the standalone performance of SnS_2_.^21,22^ This limitation arises from the low electrical conductivity and moisture-induced surface adsorption effects of SnS_2_, which slow down gas interaction and recovery processes.^14,23^ Combining SnS_2_ with conductive MXenes has become very popular in this area. MXenes have a lot of active sites, high carrier mobility, and a strong ability to adsorb gas. This makes MXene-based composites attractive for room-temperature ammonia detection because they can transfer charge faster and are more sensitive.^24^

MXene-based composites often encounter challenges in humid environments, where water molecules can strongly interact with the sensing surface. Such humidity effects can influence ammonia adsorption–desorption dynamics and, consequently, affect real-time NH_3_ detection performance under ambient conditions. In order to mitigate this issue, various strategies have been employed to regulate the impact of humidity, including plasma treatment, the application of hydrophobic coatings, or the alteration of interfaces among materials.^25^ A hydrophobic surface functionalization can make composites more stable, but it can also make them more resistant and slightly less sensitive.^26^ Nafion (NF), as a functional reagent, can be a good material for controlling moisture without compromising the ability of the composite to sense ammonia gas because it has a unique balance of hydrophobic fluorocarbon chains and hydrophilic sulfonic acid groups.^27,28^ A thin layer of Nafion protects the material from moisture and provides ammonia with abundant adsorption sites, which makes sensors more stable and sensitive for real-time and IoT use.

In the present work, a comparative sensing efficiency analysis of multilayered and few-layered MXene-based nanohybrid composites was conducted for NH_3_ sensing. The experimental results revealed that the few-layered MXene-based composite produced a higher response to NH_3_. The higher sensing response was due to the greater number of active sites, larger surface area, and higher conductivity of the few-layered MXene. The composite based on the few-layered Ti_3_C_2_T_*x*_ in combination with the flower-like SnS_2_ forms effective diffusion channels and improves gas exchange and charge movement. In order to improve selectivity and stable performance under humid conditions, the composite was functionalized with NF. The sulfonic acids in NF react with the oxide and hydroxyl groups on SnS_2_ and MXene to form acid–base interactions with NH_3_, and the sequential presence of a fluorocarbon backbone on NF prevents interference with moisture. Owing to these effects, the few-layered Ti_3_C_2_T_*x*_/SnS_2_/NF is a highly favorable nanohybrid composite for the real-world applications of stable, selective, and highly sensitive NH_3_ detection at room temperature.

## Materials and methods

### Materials

All the chemicals utilized in this research were of the highest quality and did not need further purification. We obtained titanium (about 300 mesh, 99.99%) and carbon (about 5000 mesh, 99.95%) powders from Shanghai Macklin Co., Ltd. Aluminum (about 500 mesh, 99.95%), hydrofluoric acid (≥40%, AR), and tetramethylammonium hydroxide (TMAOH) were sourced from Shanghai Aladdin Co., Ltd. We acquired SnCl_4_·5H_2_O from Sigma Aldrich and obtained thiourea (99%) from Aojin Chemical. Finally, citric acid anhydrous (99%) and Nafion (5 wt%) were obtained from Shanghai Macklin Co., Ltd.

### Preparation of MAX

Elemental powders of Ti, Al, and C in a molar ratio of 3.0 : 1.1 : 1.9 were used for synthesizing the MAX phases. The mixed powders were subjected to planetary ball milling using yttria-stabilized zirconia balls at a 5 : 1 ball-to-powder ratio, rotating at 300 rpm for 16 hours to ensure uniform mixing. The resulting homogeneous mixture was then cold-pressed under 20 MPa into compact discs and placed into alumina crucibles for sintering. In a tube furnace with flowing argon gas, the samples were sintered at a rate of 5 °C per minute until the temperature reached 1500 °C, followed by a hold of 3 hours. Once cooled to room temperature, the sintered material was obtained and processed into fine powder by grinding and sieving through a 325-mesh sieve to produce uniform MAX phase powder.

### Preparation of MXene

To start the etching process, 2 g of the MAX phase powder was slowly added to 40 mL of 40% hydrofluoric acid (HF) and magnetically stirred at 40 °C for 24 hours. After etching, the suspension was diluted with deionized water and transferred to a 50-mL centrifuge tube. It was centrifuged for 5 minutes in order to separate the supernatant. This step was repeated 5–6 times until the pH of the supernatant exceeded 6. We placed the remaining solid in a vacuum oven at 60 °C for overnight drying to obtain the multilayered MXene powder. In the delamination process, 0.5 g of the multilayered MXene powder was mixed with 10 mL of a 10 wt% TMAOH solution and stirred at room temperature for 4 hours. After 4 hours, the solution was centrifuged at 8000 rpm for 5 min, and the resulting solid particles were washed several times using deionized water until the pH was <8. Following this, the resulting powder was ultrasonicated for 1 hour under argon to help facilitate exfoliation. The resulting dark suspension was centrifuged again and subsequently freeze-dried to obtain few-layered MXene sheets.

### Preparation of flower-shaped SnS_2_

The fabrication of a flower-like SnS_2_ nanostructure was carried out *via* a simple solvothermal method. A total of 225.63 mg of SnCl_4_·5H_2_O, 228.36 mg of thiourea, and 210.14 mg of citric acid (CA) were added gradually to ethylene glycol under magnetic stirring and stirred for 60 min to obtain a uniform mixture. The as-prepared mixture solution was transferred to a 100-mL Teflon-lined stainless-steel autoclave and heated to 190 °C for 18 hours. Following the reaction, the Teflon-lined autoclave was allowed to cool to room temperature. The product was centrifuged at 6000 rpm and washed five consecutive times with an aqueous solution of deionized water and ethanol to remove unreacted precursors/impurities. Finally, the product was dried in the oven at 60 °C overnight to obtain the flower-like SnS_2_ powder.

### Preparation of Nafion-functionalized multi/few-layered Ti_3_C_2_T_*x*_/SnS_2_ nanohybrid composite

**Fig. 1:**
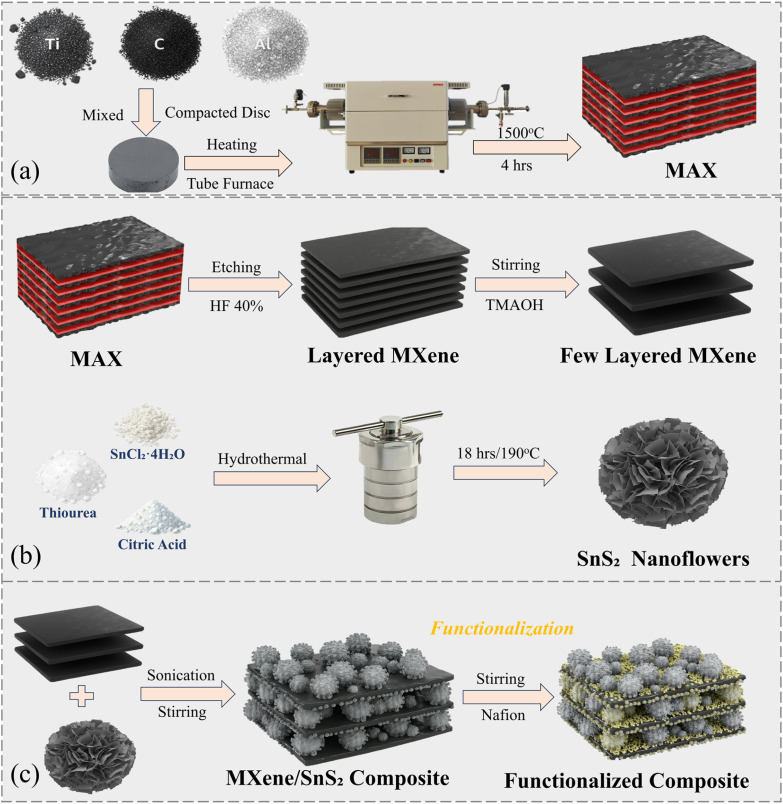
Schematic of the process adopted to prepare the (a) MAX, (b) few-layered Ti_3_C_2_T_*x*_ MXene and SnS_2_ nanoflower, and (c) few-layered Ti_3_C_2_T_*x*_/SnS_2_ nanoflower composite.

The MXene/SnS_2_ composite was prepared separately after the synthesis of SnS_2_ to avoid MXene oxidation, since the high temperature (∼190 °C) used during SnS_2_ formation could damage its surface. The adopted process for fabrication is illustrated in Fig. 1. For every composite, we placed MXene and flower-like SnS_2_ in 20 mL of deionized (DI) water separately and used ultrasonication for 15 minutes to make sure that they were evenly mixed. The sonicated SnS_2_ suspension was then slowly mixed with the predetermined amount of MXene (10, 20, and 30 wt%).^29^ For the few-layered MXene composites, lower concentrations of MXene (8, 10, and 12 wt%) were used because the delaminated layers provide a larger surface area, allowing effective interaction even at reduced loadings.^30,31^ The mixture was then ultrasonicated for 3 hours at room temperature to facilitate the even mixing of the MXene/SnS_2_ mixtures. The mixture was further stirred at room temperature for 1 hour and then centrifuged at 7000 rpm 2–3 times to prevent large aggregates. The collected precipitates were dried overnight at 60 °C to obtain fine composite powder. Three of the composites containing multilayered Ti_3_C_2_T_*x*_ were labeled Comp-10L, Comp-20L, and Comp-30L. The composites with few-layered MXenes were labeled Comp-8, Comp-10, and Comp-12, according to their MXene contents of 8, 10, and 12 wt%, respectively.

To make the composite more selective for ammonia and less affected by moisture, the few-layered MXene/SnS_2_ composite was functionalized with NF. In this study, the NF concentration was optimized to form a thin functional layer that balances humidity tolerance with gas accessibility. An optimal NF loading is known to reduce excessive water uptake and suppress the bulk swelling of NF in high-humidity environments while preserving ionic selectivity. Therefore, three different suspensions were prepared, each containing 80 mg of the composite in 80 : 20 v/v ethanol/deionized water, and ultrasonicated for 15 minutes. After sonication, the suspensions were stirred at room temperature for 1 hour. Then, NF solution was added dropwise in amounts of 0.1, 0.2, and 0.3 mL to the different suspensions that were being stirred.^23^ Each suspension was stirred for another 1 hour at room temperature. The samples were uniformly mixed, centrifuged, washed with deionized water 2–3 times, and dried at 60 °C for 6 hours. Finally, the NF-functionalized products were labeled Comp-10 NF@0.1, Comp-10 NF@0.2, and Comp-10 NF@0.3, according to their concentrations of NF.

### Characterizations

Through X-ray diffraction (XRD, Bruker D8 ADVANCE), we obtained crystallographic parameters. We then analyzed the types of elements in our samples *via* X-ray photoelectron spectroscopy (XPS, Thermo Scientific ESCALAB 250Xi). The composition and morphology of the samples were then analyzed through field-emission transmission electron microscopy (TEM, FEI Tecnai G2 F30) and field-emission scanning electron microscopy (SEM, FEI INSPECT F50). We examined the chemical bonds of the synthesized composite and functional groups using Fourier-transform infrared (FTIR) spectroscopy.

### Gas sensing

The gas-sensing performance of the prepared samples was evaluated using a modified Sino-AGG Tech AES-4SD Flexible Device Analysis System. The sensing measurements were conducted using silver interdigitated electrodes on an alumina ceramic substrate. In order to prepare the film, 100 mg of the sensing material was dissolved in 1 mL of ethanol and ultrasonically dissolved to obtain a uniform suspension. The resulting paste was drop-cast onto the electrode surface and dried under vacuum at 60 °C for 3 hours. The sensing measurements were performed at a constant applied voltage, where the sensors were exposed to various target gases, including NH_3_, CO_2_, ethanol, acetone, trimethylamine, and formaldehyde. For baseline comparison under typical indoor dry-air conditions, the response was recorded at 25 °C and 35% relative humidity (RH). The sensor response (*S*_h_) was calculated as the ratio of resistance in the target gas (*R*_g_) to that in air (*R*_a_).
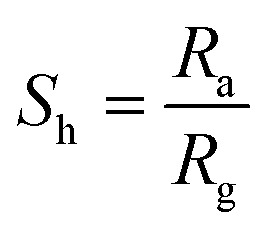


When the gas is absorbed, it produces a response for the gas through a resistance change. The response time of the sensor is the amount of time it takes for 90% of the total resistance to change. When gas is desorbed, the recovery time is the amount of time it takes to return to 90% of the baseline resistance.

## Results and discussion

**Fig. 2:**
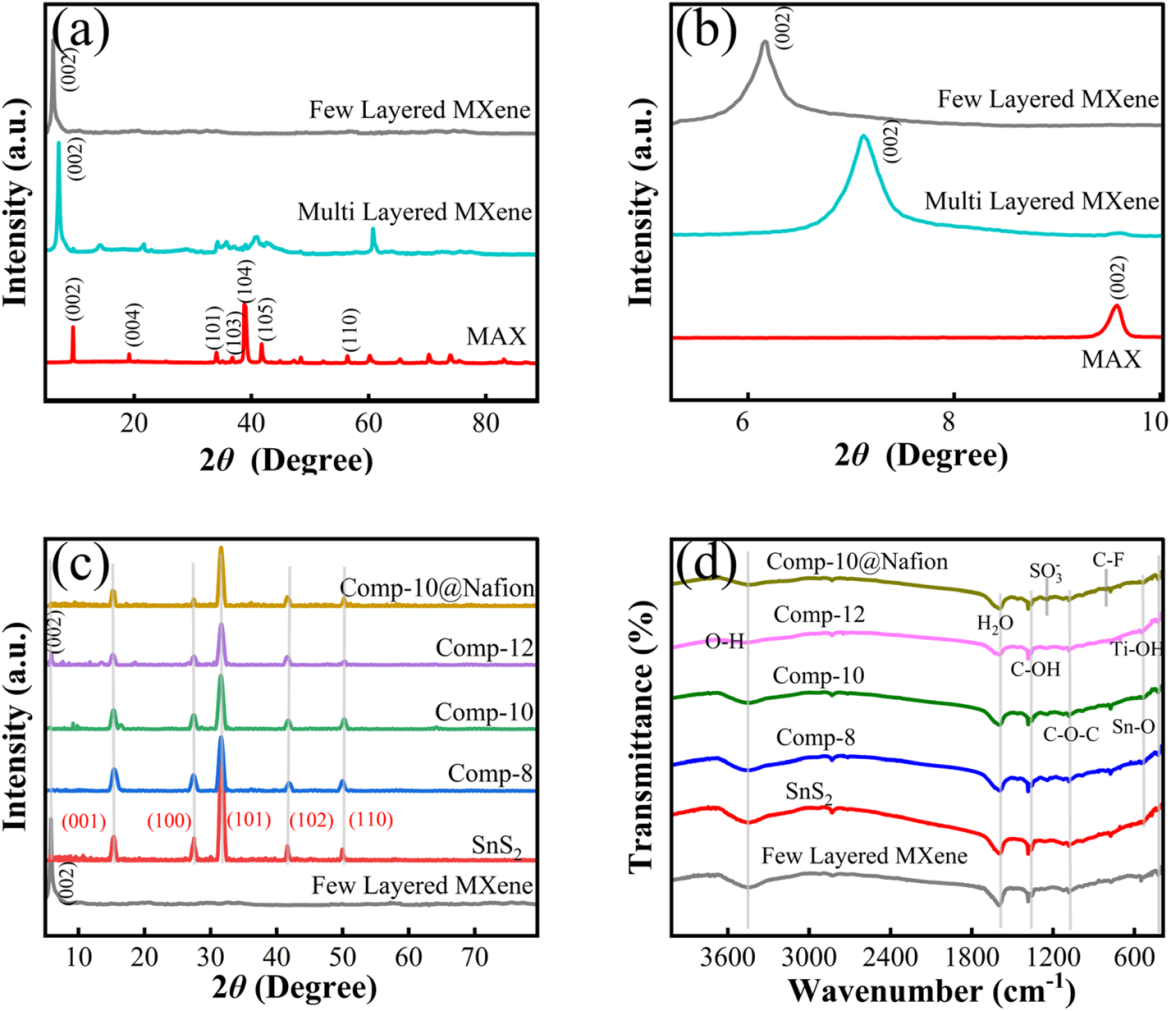
(a) XRD pattern of MAX and multilayered and few-layered MXene. (b) Low-angle XRD pattern of MAX and MXenes. (c) XRD pattern of few-layered MXene, SnS_2_, all composites, and the NF-functionalized composite. (d) FTIR spectra of few-layered MXene, SnS_2_, all composites, and the NF-functionalized composite.

The crystalline behavior of the MAX phase, Ti_3_C_2_T_*x*_ nanosheets, and all composites was examined through XRD. After etching the MAX, the (104) peak around 2*θ* ≈ 38.43° completely disappeared, and the diffraction peaks between 33° and 43° became much weaker than those of Ti_3_AlC_2_. This change indicates that aluminum was successfully etched away from the MAX phase. Furthermore, the (002) peak of Ti_3_C_2_T_*x*_ shifted to a lower angle, *i.e.* 7.21°, as shown in Fig. 2(a). These shifts suggest a reduction in structural order and an increase in the spacing between layers, both of which are typical signs of multilayered MXene formation.^32^ The delamination treatment of multilayered MXene into few-layered sheets made the (002) peak the most intense and dominant feature in the XRD pattern, shifting it further to 6.19°, as shown in the low-angle XRD pattern in Fig. 2(b). The shift to a lower angle corresponds directly with an increase in the interlayer spacing between the MXene sheets. This is an indication of the delamination process, where the gap between the layers expands as the multilayered structure exfoliates.^33^ In the low-angle XRD region, the prominent (002) reflection observed at 6.19° suggests that the delamination process effectively expanded the interlayer distance between the MXene sheets, confirming the formation of few-layered MXene structures. This shift provides valuable insight into the structural changes induced by the delamination treatment. The XRD profile clearly confirmed the successful synthesis of the MAX and few-layered MXene after etching and delamination. The XRD pattern of the SnS_2_ nanoflowers displayed distinct peaks at 15.3°, 28.03°, 32.02°, 41.8°, and 50.4°, as shown in Fig. 2(c). These diffraction peaks correspond to the (001), (100), (101), (102), and (110) planes of SnS_2_.^14,34^ When the few-layered Ti_3_C_2_T_*x*_ MXene was integrated with the SnS_2_ nanoflowers to form the nanohybrid composite samples (Comp-8, Comp-10, and Comp-12), noticeable variations appeared in their XRD patterns. Among these, only Comp-12 exhibited the characteristic diffraction peaks of MXene at 2*θ* = 6.19°, which correspond to the (002) plane. The lack of MXene peaks in Comp-8 and Comp-10 is probably because of their lower MXene content. Fig. 2(c) shows the XRD pattern of Comp-10 after the addition of 0.2 mL of NF solution. The diffraction peaks are exactly the same as those of the original Comp-10. This means that adding NF does not produce any structural changes in the nanohybrid composite.^27^ These results confirm the successful integration of few-layered Ti_3_C_2_T_*x*_ within the SnS_2_ framework. They also highlight that the detectability of MXene-related reflections in the XRD patterns is directly dependent on the amount of MXene in the composite.^14^

The Fourier transform infrared (FTIR) spectra of the prepared samples are shown in Fig. 2(d). The absorption bands appearing at 3812 and 1356 cm^−1^ correspond to the vibrations of hydroxyl groups and molecularly adsorbed water (–C–O–H), respectively. As the proportion of MXene increases and the amount of SnS_2_ decreases, the intensity of these bands gradually decreases, suggesting a reduced presence of surface hydroxyl species. The feature observed at 1082 cm^−1^ is attributed to the stretching vibration of the C–O–C bond.^35,36^ The low-wavenumber absorptions at 422 and 538 cm^−1^ are associated with Ti–OH and Sn–O vibrations, respectively.^37,38^ After NF functionalization, two new absorption peaks appear at 1246 cm^−1^ and 790 cm^−1^, which can be attributed to the symmetric stretching of the sulfonate group (–SO_3_^−^) and the C–F stretching vibration from the fluorinated NF backbone, respectively.^39^ These additional peaks clearly confirm the successful functionalization of NF with the few-layered Ti_3_C_2_T_*x*_/SnS_2_ composite surface. Although the overall peak intensities slightly decrease, the main spectral features of SnS_2_ and MXene remain visible, indicating that NF forms a thin and uniform coating without altering the core structure of the nanohybrid. In summary, the FTIR analysis demonstrates the consistent evolution of the structure of the MAX phase to MXene and later on to the SnS_2_-based composites. The NF-functionalized composite exhibits extra sulfonate and fluorocarbon characteristics, which validate good surface modification without a compromise in the structural integrity or typical bonding arrangement of the composite.

**Fig. 3:**
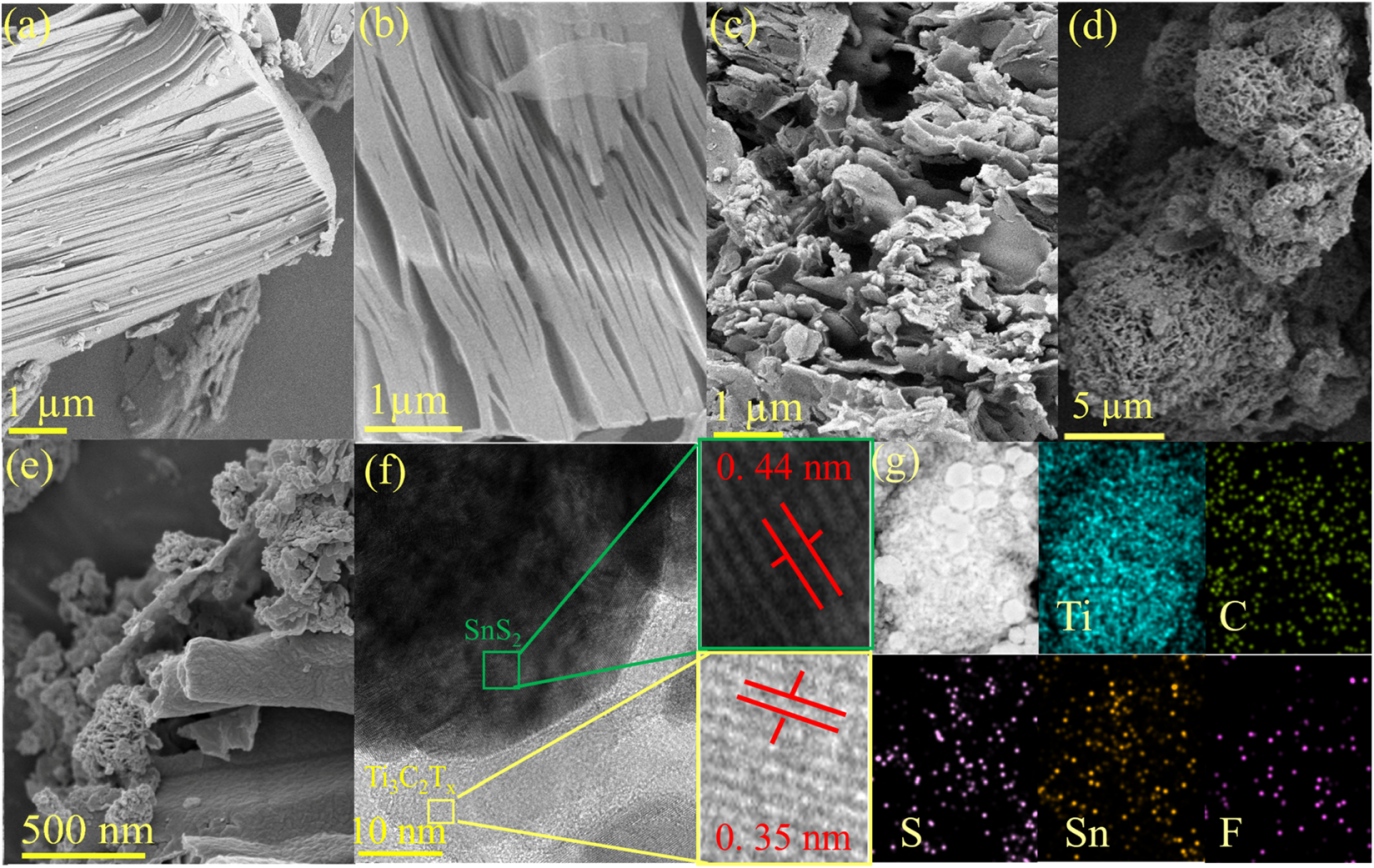
(a) SEM image of MAX, (b) layered MXene, (c) few-layered MXene, and (d) SnS_2_ nanoflowers. (e) SEM image of SnS_2_ nanoflowers attached to the surface of few-layered MXene. (f) High-resolution TEM image of the composite. (g) TEM elemental mapping of Ti, C, Sn, S, F and O.

Additionally, scanning electron microscopy (SEM) and high-resolution transmission electron microscopy (HRTEM) were used to study the microstructure and morphology of the gas-sensitive materials. The structure of the MAX phase is confirmed by the SEM images shown in Fig. 3(a), and the MXene produced by HF etching is shown in Fig. 3(b). Fig. 3(c) shows the obtained few-layered MXene after further delamination with the TMAOH solution. The individual sheets are well-separated and uniformly distributed, confirming successful exfoliation. Fig. 3(d) clearly displays the flower-like morphology of SnS_2_, providing abundant active sites and a high surface-to-volume ratio, both of which are very beneficial for gas adsorption and sensing processes. Fig. 3(e) shows that the SnS_2_ nanoflowers are attached to the surfaces of the MXene layers. This SEM image is the evidence of the MXene/SnS_2_ composite system that was already validated by XRD and FTIR. Fig. 3(f) shows the HRTEM image of the MXene sheets with SnS_2_ nanoflowers attached to their surface. The MXene sheets exhibit a fringe spacing of 0.35 nm, corresponding to the (002) plane, indicating exfoliated few-layered MXene. The SnS_2_ nanoflowers show a larger fringe spacing of 0.44 nm, attributed to the (001) plane of SnS_2_, reflecting the interlayer distance of the material. The observed difference in fringe spacing between MXene and SnS_2_ highlights their distinct structural characteristics. The TEM mapping profile {Fig. 3(g)} confirms the existence of all individual constituent elements in the composite. Additionally, the images demonstrate a uniform distribution of all the elements, which confirms the formation of a composite system.

**Fig. 4:**
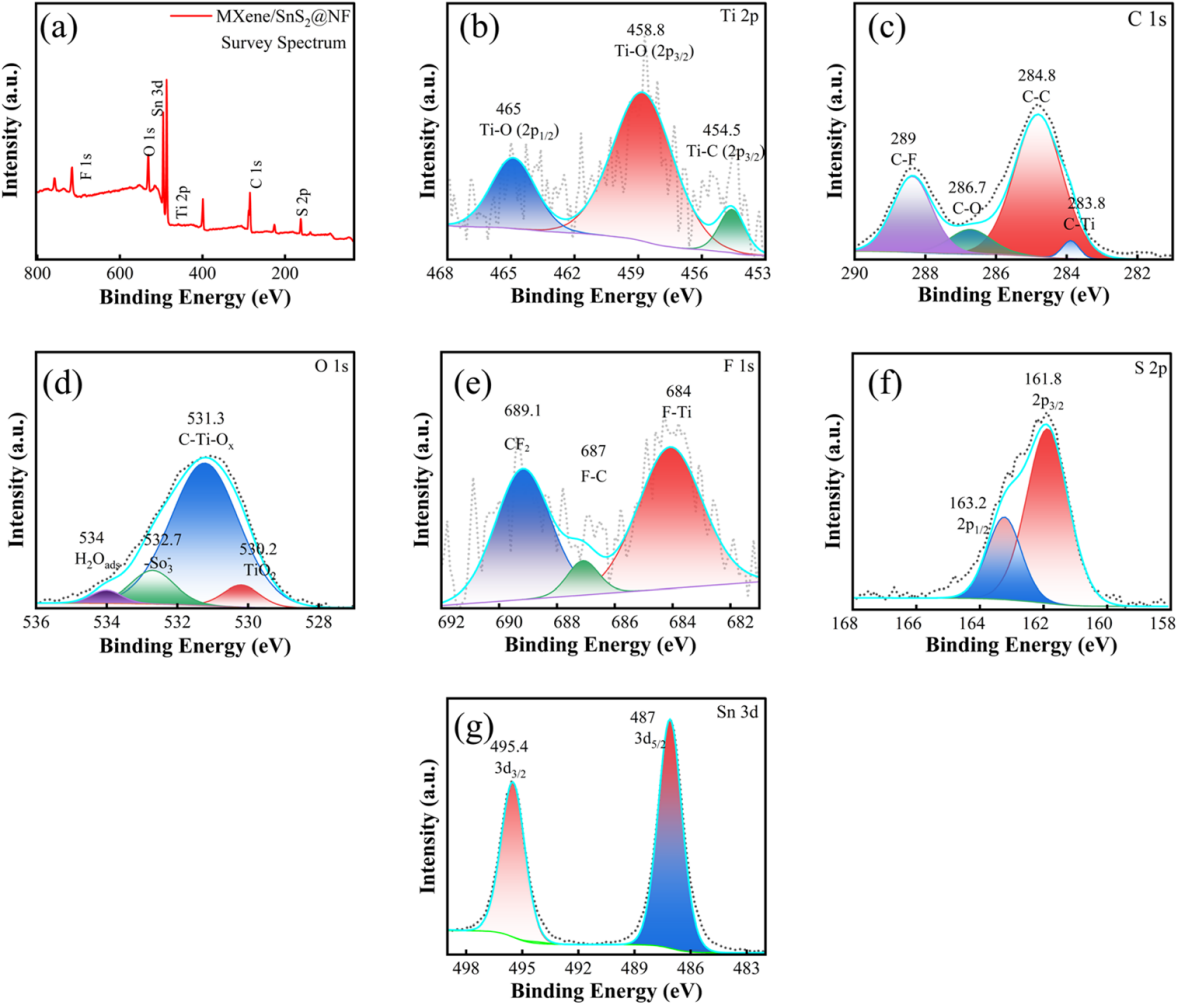
XPS analysis of the NF-functionalized few-layered Ti_3_C_2_T_*x*_/SnS_2_ composite: (a) survey spectrum, (b) Ti 2p spectrum, (c) C 1s spectrum, (d) O 1s spectrum, (e) F 1s spectrum, (f) S 2p spectrum, and (g) Sn 3d spectrum of the functionalized composite.

XPS was employed to obtain further insight into the finer details of the elemental oxidation states of the composites. Fig. 4(a) shows the full elemental spectrum of the composite, making it clear that all the elements are present. Fig. 4(b) shows the Ti 2p spectrum, which can be resolved into three separate peaks. The peak at 454.5 eV is assigned to the Ti–C bonds that are typical of MXene. The peaks at 458.8 eV and 465 eV are assigned to the Ti–O bonds.^40^Fig. 4(c) shows the C 1s spectrum of the Ti_3_C_2_T_*x*_/SnS_2_/NF composite, with peaks at 283.8, 284.8, 286.7, and 289 eV. These correspond to Ti–C bonds, neutral carbon (C–C), oxygen-containing groups (C–O), and fluorine-bound carbon (C–F), respectively.^41^ The C–C peak reflects unoxidized carbon on the surface, the C–O peak indicates the presence of MXene surface terminations,^12^ and the C–F peak confirms that NF has been successfully functionalized onto the composite. The O 1s spectrum of the Ti_3_C_2_T_*x*_/SnS_2_/NF composite {Fig. 4(d)} shows peaks at 530.2, 531.3, 532.7, and 534.0 eV, corresponding to TiO_2_, C–Ti–O_*x*_ surface terminations,^11^ –SO_3_^−^ groups from NF,^42^ and adsorbed water, respectively. These peaks confirm partial MXene oxidation, the presence of functional groups, and successful NF functionalization. The F 1s spectrum in Fig. 4(e) shows peaks at 684.0, 687.0, and 689.1 eV, corresponding to F–Ti, F–C, and –CF_2_ bonds, respectively. The F–Ti and F–C signals reflect fluorine bonded to titanium and carbon in the MXene, respectively, while the –CF_2_ peak arises from fluorocarbon groups, further confirming surface functionalization.^43^ The S 2p peaks at 161.8 and 163.2 eV correspond to S 2p_3/2_ and S 2p_1/2_, respectively, and are shown in Fig. 4(f),^44^ while the Sn 3d peaks in Fig. 4(g) at 487.0 and 495.4 eV confirm the presence of SnS_2_.^45^ These findings, together with other spectra, confirm successful composite formation and surface functionalization.

### Sensing performance evaluation

**Fig. 5:**
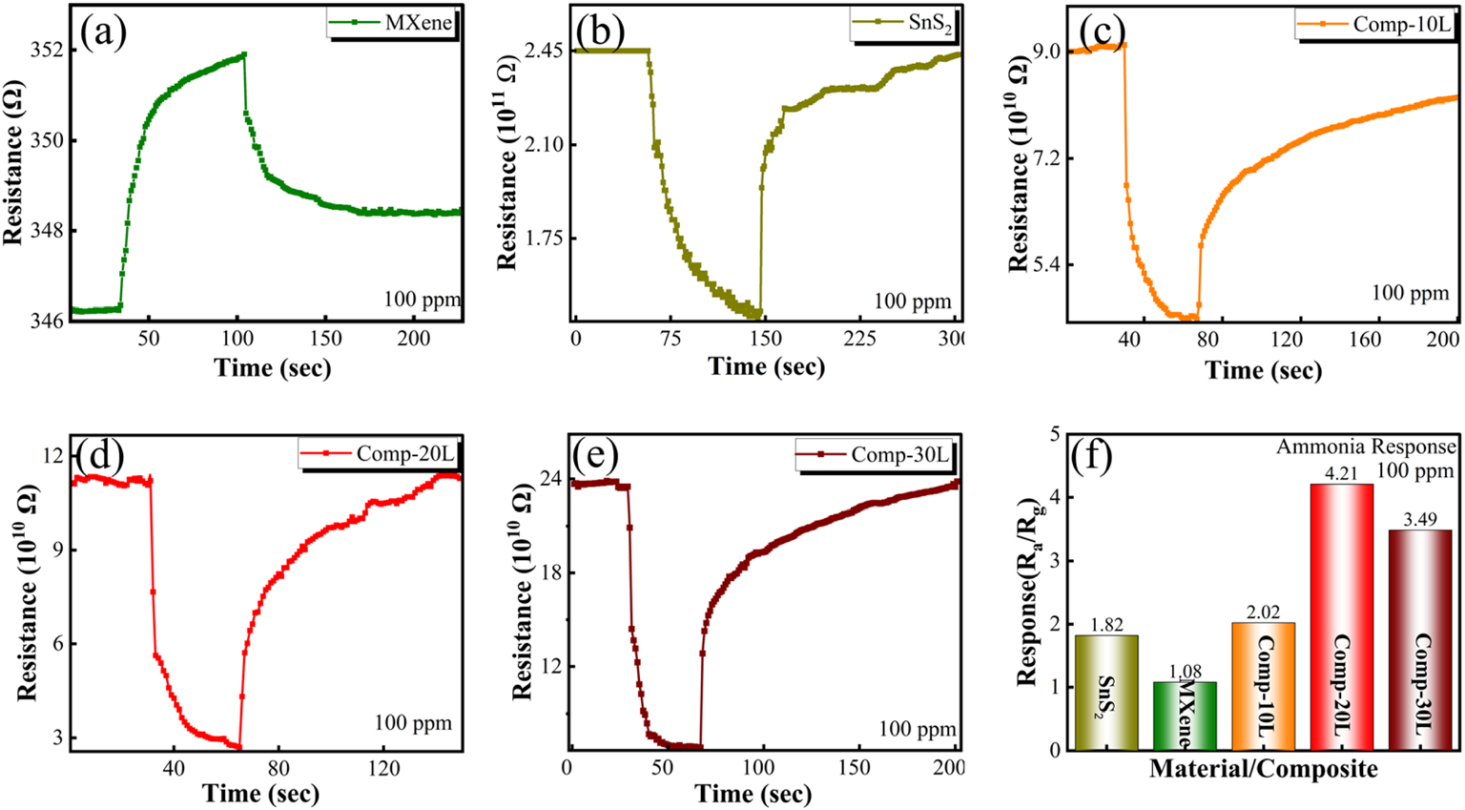
Base resistance and change in resistance of (a) multilayered MXene, (b) SnS_2_, (c) the composite of multilayered MXene/SnS_2_ nanoflowers, *i.e.* Comp-10L, (d) Comp-20L, and (e) Comp-30L upon exposure to 100 ppm ammonia. (f) Response of all materials and composites to 100 ppm ammonia.

A two-probe resistive sensor was evaluated at room temperature for the detection of NH_3_. We measured and recorded the changes in electrical resistance when exposing the sensor to ammonia and air at a constant bias voltage. The sensor was tested at 25 °C and 35% RH as a standard for normal indoor air conditions with dry air. To define the baseline resistance, we measured the response of each constituent material to ammonia, which is presented in Fig. 5. Multilayered MXene had an intrinsically low electrical resistance and exhibited a p-type sensing behavior. The transport characteristics of the materials were identified from their intrinsic electronic properties and gas-response behavior. For example, the MXene showed an increase in resistance when it was exposed to 100-ppm ammonia, as shown in Fig. 5(a), which is a commonly reported behavior for MXene-based sensors.^29,37^ Ti_3_C_2_T_*x*_ MXene exhibited p-type behavior, meaning it conducted electricity through holes (positive charge carriers). This behavior is commonly associated with the presence of surface functional groups, such as –O and –OH, which influence charge transport and lead to hole-dominated conduction characteristics. When exposed to NH_3_, the ammonia molecules donated electrons, filling the holes and increasing the resistance, which is typical for p-type materials. When the SnS_2_-based sensor was exposed to 100-ppm ammonia, its resistance decreased, as shown in Fig. 5(b). The observed reduction in resistance represents an n-type behavior of SnS_2_, where adsorbed NH_3_ molecules donate electrons to the material with electrons as the majority charge carriers, increasing its overall charge density and making it more electrically conductive. In order to better understand the interfacial effects and fundamental synergy between the multilayered/few-layered MXene and flower-shaped SnS_2_, their composites were examined for ammonia sensing. Initially, the multilayered MXene/SnS_2_ composites were synthesized to analyze their gas-sensing performance. Three composites, designated as Comp-10L, Comp-20L, and Comp-30L, were prepared by varying the MXene content. The resistance of these composites decreased upon exposure to 100-ppm ammonia {Fig. 5(c)–(e)}, revealing an n-type sensing behavior, mainly due to the dominant involvement of SnS_2_ in the composite. As shown in Fig. 5(f), Comp-20L demonstrated the highest response ratio of 4.21, followed by Comp-30L and Comp-10L.

**Fig. 6:**
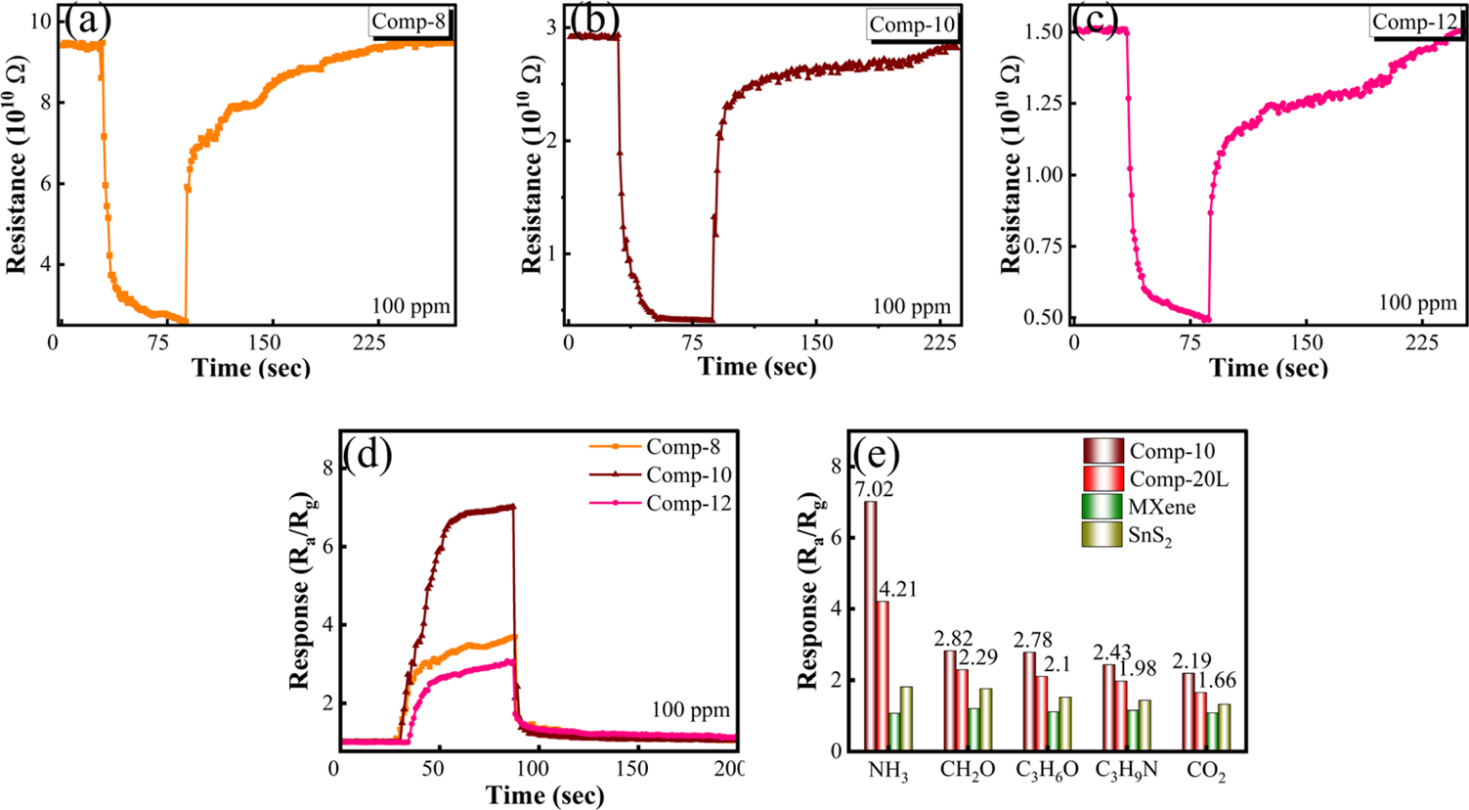
Base resistance and change in resistance of (a) the composite of few-layered MXene/SnS_2_ nanoflowers, *i.e.* Comp-8, (b) Comp-10, and (c) Comp-12 upon exposure to 100 ppm ammonia. (d) Response of all composites to 100 ppm ammonia. (e) Response of few-layered MXene/SnS_2_ nanoflowers and multilayered MXene/SnS_2_ nanoflowers, MXene and SnS_2_ to 100 ppm ammonia and other interfering gases.

To compare the sensing performance of multilayered and few-layered MXene/SnS_2_ composites and to determine which configuration provides enhanced ammonia detection, few-layered MXene/SnS_2_ nanoflower composites were synthesized, and their response to ammonia was investigated. Three samples (named Comp-8, Comp-10, and Comp-12 based on their MXene content) were tested at room temperature with 100-ppm ammonia. Upon exposure, all the composites showed a clear drop in resistance {Fig. 6(a–c)}, confirming their n-type sensing characteristics. As shown in Fig. 6(d), the response intensity varied with the MXene concentration. Comp-10 had the highest response value of 7.02 when exposed to 100-ppm ammonia. This response value was much higher than those of its counterparts. Therefore, these findings indicate that the Comp-10 ratio is the most effective formulation for maximizing ammonia detection. The results also show that the few-layered MXene combined with SnS_2_ nanoflowers has better charge transfer pathways and better surface interactions than the multilayered MXene configuration. The selectivity of Comp-10 was also examined by exposing it to several common environmental gases at 100 ppm {Fig. 6(e)}, including formaldehyde, acetone, trimethylamine, and carbon dioxide. We compared these results with those of the optimized composite of multilayered MXene and individual constituent materials to get a better understanding of the selectivity of all the materials and composites. Although our few-layered MXene-based sensor reacted the strongest with ammonia, it also showed a noticeable cross-sensitivity for other common interfering gases. It is very common for metal sulfide and carbon-based sensors to show a lack of selectivity at room temperature.^46^ This cross-sensitivity occurs in these materials because many gases similarly donate electrons to the sensor. Materials like few-layered MXene also promote non-specific gas reactions because of their high surface area.^47^ For practical implementation, this is a major challenge, since the sensor must suppress interference from coexisting gases for reliable ammonia detection in complex environments.

**Fig. 7:**
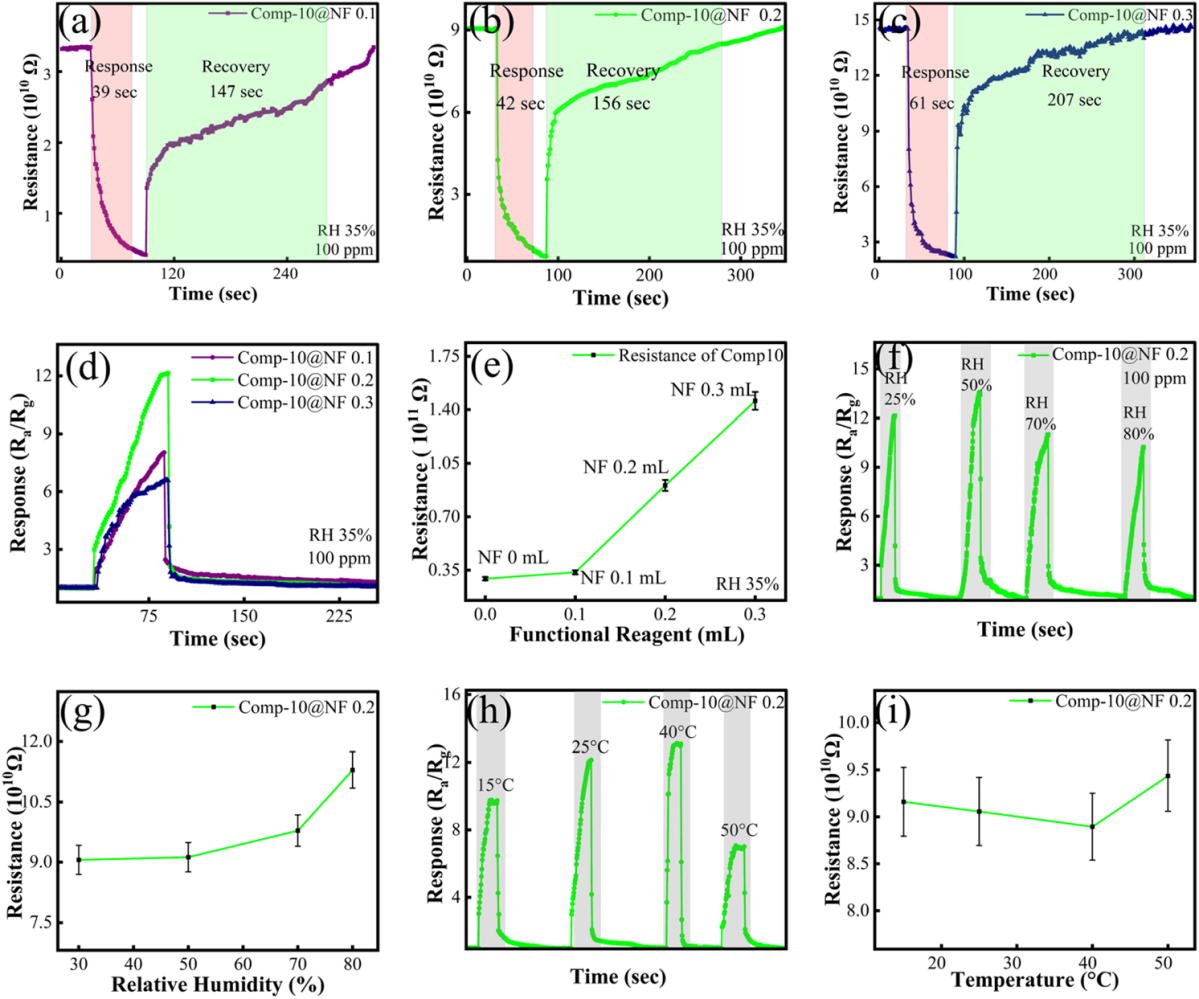
Base resistance and change in resistance of the optimized composite of few-layered MXene/SnS_2_ nanoflowers, *i.e.* Comp-10, with varying NF concentrations of (a) 0.1, (b) 0.2, and (c) 0.3 mL, upon exposure to 100 ppm ammonia. (d) Response of all functionalized composites of Comp-10 with NF. (e) Change in base resistance of the optimized composite, *i.e.* Comp-10, with varying NF concentrations. (f) Effect of RH on the response and (g) baseline resistance of the sensor. (h) Effect of temperature on the response and (i) baseline resistance of the sensor.

An NF solution was used to functionalize the sensor to make it more selective to ammonia. The concentration of NF was optimized to ensure the best performance, as excessive surface functionalization could block active sites, increase overall resistance, and potentially lead to swelling under high-humidity conditions. We tested three concentrations: 0.1 mL, 0.2 mL, and 0.3 mL. The response values for all the functionalized composites are shown in Fig. 7(d). At a 0.1-mL concentration, the response was only 8.03 {Fig. 7(a)}, showing a small increase compared to the unfunctionalized sensor. The results revealed that 0.2 mL was the most effective concentration, yielding the highest response of 12.11 against 100-ppm NH_3_, with response and recovery times of 42 s and 156 s, respectively {Fig. 7(b)}. The response dropped significantly to 6.58 when the NF concentration was increased to 0.3 mL, as shown in Fig. 7(c). The presence of a moderate concentration of NF (–SO_3_H groups) enhances the conduction of protons and amplifies the sensitivity of the sensor, as well as the adsorption of ammonia. With a further increase in the concentration of NF, beyond the optimum concentration, it may block active sites and increase resistance, leading to a reduced response. Besides maximizing the response magnitude, the NF concentration also had an effect on the kinetic performance of the sensor. As shown in Fig. 7(c), the higher concentration of 0.3 mL significantly increased the recovery time, while the response time increased marginally. The thicker NF layer traps more ammonia molecules, which makes it take longer to recover because the stronger binding interactions require more energy to desorb the gas.^23^ In addition to acting as a gas diffusion barrier that physically hinders gas adsorption and desorption, higher concentrations of NF also have a large impact on the electrical conductivity of the composite material. Fig. 7(e) shows that the baseline resistance of the sensor increased gradually with the NF concentration, reaching its maximum value at 0.3 mL of NF. This happens because the sulfonic acid (SO_3_H) groups and fluorocarbons form thicker insulating layers that stop the flow of electrons. In summary, slower ammonia desorption and increased electrical resistance led to a weaker sensing signal and slower response/recovery at higher NF concentrations. Therefore, optimizing the NF concentration is essential for effective composite functionalization.

Subsequent investigations were conducted to systematically evaluate the influence of environmental humidity and operating temperature on the sensing characteristics of the optimized sensor. As the RH increased from 25% to 50%, the sensor response toward NH_3_ increased, as shown in Fig. 7(f). This can be attributed to the presence of a moderate amount of adsorbed water molecules that enhance surface charge transfer and facilitate the interaction between NH_3_ and the sensing layer. NF, with its hydrophilic sulfonic groups, can retain limited moisture, promoting proton-assisted conduction and improving gas adsorption at moderate humidity levels. However, further increasing the RH beyond 50% led to a gradual decrease in response, likely due to excessive water adsorption that blocks active sensing sites and competes with NH_3_ molecules, thereby suppressing effective gas–surface interactions. Meanwhile, the baseline resistance continuously increased with the RH, as shown in Fig. 7(g), and this is associated with increased carrier scattering and reduced charge transport caused by adsorbed water layers on the sensor surface. The sensor response also showed a strong dependence on the operating temperature. At 15 °C, the response was relatively low because of limited thermal energy, which restricts NH_3_ diffusion and surface reaction kinetics, as shown in Fig. 7(h). Increasing the temperature to 40 °C significantly enhanced the response, reaching a maximum value of 13.09, as a higher temperature promotes faster adsorption–desorption dynamics and a more efficient charge transfer. However, when the temperature was further increased to 50 °C, the response decreased to 6.98, which can be attributed to the accelerated desorption of NH_3_ molecules and reduced residence time on the active sites. The baseline resistance showed a slight decrease as the temperature increased from 15 °C to 40 °C {Fig. 7(i)}, mainly due to improved charge carrier mobility and thermally activated transport. However, at temperatures above 40 °C, the baseline resistance increased, which can be attributed to the enhanced desorption of surface species and partial suppression of NF-assisted proton conduction, resulting in reduced charge transfer efficiency. Overall, the results confirm that the sensor sustains a consistent ammonia response and manageable baseline variation under changing humidity and temperature conditions, underscoring its robustness for practical sensing applications.

**Fig. 8:**
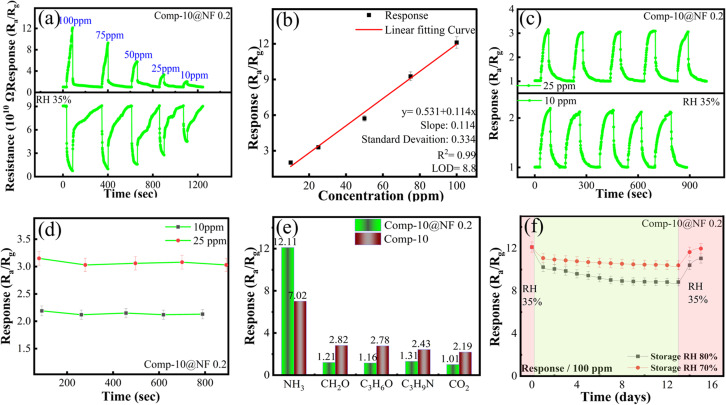
(a) Reproducibility testing of 0.2 mL Comp-10@NF of 100–10 ppm. (b) Correlation of the response of the sensor with ammonia concentration of 100–10 ppm. (c) Repeatability testing across 25 and 10 ppm concentration. (d) Error bars showing the response variability across each cycle. (e) Response to ammonia and other interfering gases, including comparison with the unfunctionalized Comp-10. (f) Reliability testing at 70% and 80% RH over a period of 12 days.

Table 1 summarizes a comparison of the sensing performance of our NF-functionalized MXene/SnS_2_ sensor with recently reported NH_3_ sensors. As shown, our sensor exhibits competitive response values and faster response times compared with most of the reported sensors. In order to investigate the reproducibility and determine the minimum ammonia concentration at which the sensor responds, the NF-optimized sensor was tested over a range of ammonia concentrations from 10 ppm to 100 ppm. Fig. 8(a) shows that the sensor produced a significant response even at low concentrations, with responses of 2.01 and 3.28 at 10 ppm and 25 ppm, respectively. Notably, 10 ppm represents the lowest NH_3_ concentration at which a reliable response was recorded under the present experimental conditions. The sensor response exhibited a clear linear trend between 10 and 100 ppm, with no saturation observed. The fitting curves for the sensor's response to NH_3_ concentration showed strong linear correlations, with an *R*^2^ value of 0.99. Using these linear fits, we calculated the theoretical limit of detection (LOD) as 8.8 ppm, as shown in Fig. 8(b). This was determined by dividing the standard deviation (*s*) of the sensor noise by the slope (*s*) of the curve three times (LOD = 3*σ*/*s*). In order to further verify the sensor's practical reproducibility, we conducted five consecutive air/NH_3_ exposure cycles against 10- and 25-ppm NH_3_ {Fig. 8(c)}. The sensor exhibited excellent reproducibility over repeated cycles, showing only minor fluctuations in the response, as shown in Fig. 8(d). This stable behavior demonstrates its high efficiency and reliable operational stability under repeated NH_3_ exposure. A comparative analysis presented in Fig. 8(e) indicates that the NF-functionalized sensor displayed excellent selectivity towards ammonia. Compared with its unfunctionalized counterpart, the functionalized sensor exhibited significantly higher sensitivity to ammonia than to other interfering gases, including formaldehyde, acetone, trimethylamine, and carbon dioxide. These results confirm that the NF coating imparts strong ammonia detection selectivity. The long-term stability of the sensor was examined under high-humidity conditions to verify its reliability for practical applications, as illustrated in Fig. 8(f). Two optimized sensors were stored under controlled conditions at relative humidity levels of 70% and 80%, while the temperature was maintained at 25 °C for a period of twelve days. The sensing response was measured every 24 hours to evaluate performance stability over time. After 12 days of storage under controlled humidity conditions, the sensor stored at 70% RH responded to 100-ppm ammonia with a response of 11.08, whereas the sensor stored at 80% RH showed a response of 10.23. The sensors showed notable but differential recovery after a 3-day recovery period at 35% RH. The 70% RH sample returned to 11.97 (98.8% of its initial response), while the 80% RH sample recovered to 11.08 (91.5% of its initial response). As a result of a reversible hydration/dehydration cycle, the sensor stored at 70% RH largely regained its activity after 3 days of recovery at 35% RH. In contrast, the sensor stored at 80% RH showed slightly incomplete recovery, primarily due to excessive moisture adsorption that caused temporary surface passivation and slowed ammonia desorption. A minor effect may also come from weak competition between ammonia and the hydrophilic SO_3_H sites in NF, which can temporarily trap moisture and slightly delay recovery. However, the stable sensing performance observed over 12 days at varying RH levels indicates that the optimized NF layer preserves charge transport and gas diffusion.

**Table 1:** Comparison of the sensing capabilities of the NF-functionalized few-layered MXene/SnS_2_ sensor and other previously developed MXene/TMDs NH_3_ sensors

Material	NH_3_ (ppm)	Response time (s)	Recovery time (s)	Response (%)^a^ or *R*_a_/*R*_g_ or *R*_g_/*R*_a_	Ref.
Ti_3_C_2_T_*x*_/SnS_2_/NF	100	42	156	91.7%	This work
Ti_3_C_2_T_*x*_/MoS_2_	10	56.8	65	1.47%	48
Ti_3_C_2_T_*x*_/SnS_2_	10	161	80	42.9%	29
Nb_2_CT_*x*_/SnS_2_	100	32	78	89.3%	49
Ti_3_C_2_T_*x*_/ReS_2_	50	60	43	7.8%	50
Ti_3_C_2_T_*x*_/MoS_2_	100	43.6	40.8	21.1%	51

a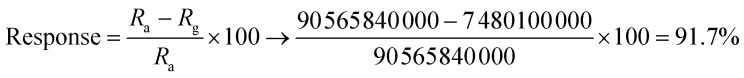.

### Sensing mechanism

The sensing performance of the NF-functionalized few-layered MXene/SnS_2_ composite arises from the synergy between surface chemistry and electronic modulation. The SO_3_H groups in NF create highly polar domains, which react intensively with ammonia by acid/base reactions, and its fluorocarbon backbone inhibits water uptake. In humid environments, NF plays a dual role. In moderate humidity levels, the hydrophilic SO_3_H groups facilitate ammonia diffusion by forming transient NH_4_^+^ species that enhance the interaction with the functional sites. This interaction not only helps in trapping ammonia molecules on the surface but also enhances the gas sensing performance of the composite, as the ammonia molecules are better adsorbed and more likely to react with the sensing surface. Increasing humidity levels can temporarily shift the baseline resistance, but the hydrophobic fluorocarbon chains of NF effectively prevent excessive moisture adsorption, allowing the sensor to maintain a repeatable and stable performance. The band structures of the composite components further enhance the sensing behavior. Ti_3_C_2_T_*x*_ MXene exhibits metallic behavior with its Fermi level in the conduction band,^52^ allowing efficient electron transfer. The Fermi level of SnS_2_, an n-type semiconductor, is higher than that of MXene, indicating a higher electron concentration.^29^ Owing to the work function difference, electrons transfer from the n-type SnS_2_ to the higher-work-function Ti_3_C_2_T_*x*_ MXene upon contact, establishing a Schottky junction. This creates an electron depletion region and upward band bending in SnS_2_ near the interface. This configuration greatly enhances the electron exchange and resistance modulation upon gas adsorption, leading to improved sensitivity.

**Fig. 9:**
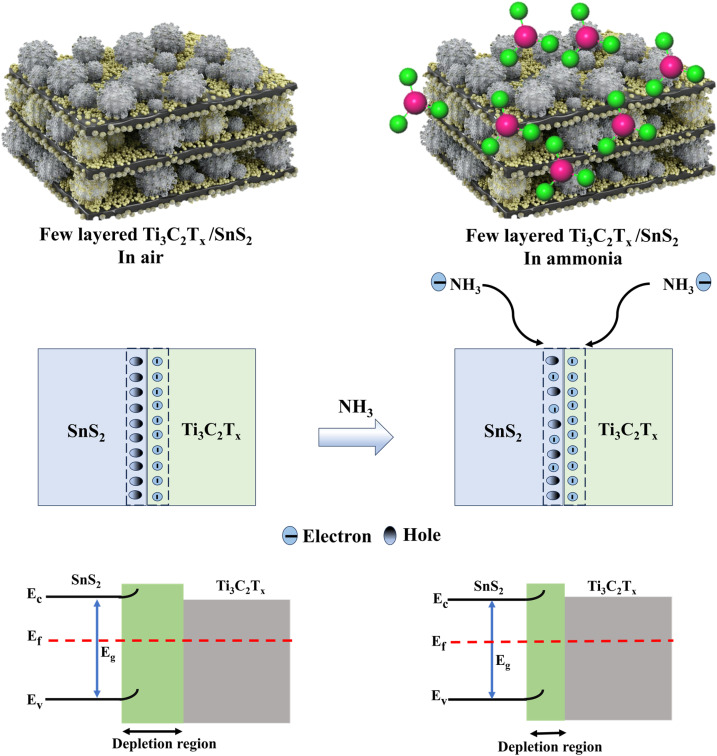
Gas sensing mechanism of the NF-functionalized Ti_3_C_2_T_*x*_/SnS_2_ sensor for ammonia, showing a schematic of the p–n junction and the conductivity pathway changes of the sensor before and after ammonia exposure.

Under ambient air conditions, oxygen molecules adsorb onto n-type SnS_2_ and extract electrons from the conduction band, resulting in chemisorbed oxygen species (O_2_^−^). The corresponding surface reaction is represented by eqn (1) and (2). This process creates an electron depletion layer near the surface and defines the baseline resistance of the sensor, as shown in Fig. 9. When ammonia is present, the strong electron-donating molecules of ammonia react at several active sites in the functionalized composite. NH_3_ forms hydrogen bonds with the sulfonic acid's SO_3_H at the NF interface and often undergoes partial proton transfer, which forms ion pairs of SO_3_^−^ and NH_4_^+^, as expressed in eqn (3).^28^ This anchoring effect keeps the ammonia molecules localized close to the surface and, hence, enables the efficient transfer of electrons to SnS_2_ domains. Furthermore, molecules of NH_3_ adsorb onto SnS_2_ at the defects and oxygen adsorption sites and give up electrons to the conduction band, as expressed in eqn (4).^29^ These donated electrons neutralize the adsorbed oxygen species, thereby reducing their electron-trapping effect and enhancing the n-type conductivity of SnS_2_. The excess electrons are subsequently transferred toward the p-type-like MXene domains at the interface, where they recombine with holes, leading to a reduction in the interfacial depletion region. As a result, the interfacial barrier height decreases, causing a significant reduction in sensor resistance. Conclusively, this synergistic interaction among NH_3_, NF, SnS_2_, and MXene enables the detection of ammonia with a high sensitivity and rapid response. The sensor also has great stability, reproducibility, and selectivity under different humidity conditions, which highlights its feasible application in the real world.

1 O_2_ (gas) → O_2_ (ads).

2 O_2_ (ads) + e^−^ → O_2_^−^ (ads).

3 SO_3_H + NH_3_ → SO_3_^−^ + NH_4_^+^.

4 2NH_3_ (gas) + 3O_2_^−^ (ads) → N_2_ + 3H_2_O + 3e^−^.

## Conclusion

The NF-functionalized few-layered MXene/SnS_2_ nanoflower sensor displays excellent room temperature ammonia detection with high sensitivity, fast response (42 s), fast recovery (156 s) and high selectivity. The final optimized composite of the few-layered MXene/SnS_2_ nanoflowers coated with 0.2 mL of NF yielded the maximum response of 12.11 to 100-ppm NH_3_. Compared with the multilayered composites, the few-layered configurations exhibit much better charge transfer and surface interactions, and NF is better at enhancing ammonia adsorption and lowering interference under humid conditions. The sensor also displayed a stable and reproducible performance even under humid conditions, which validated its robustness and effectiveness in long-term real-world applications.

## Author contributions

Waqas Saeed: investigation, conceptualization, writing – original draft, methodology, validation, writing – review and editing, formal analysis, data curation and visualization; Ye Tian: fund acquisition and supervision; Irshad Ahmad Mir: resources, visualization, validation and supervision; Baoji Miao, Amna Manzoor, Surjyakanta Rana and Xing Chen: visualization and validation.

## Conflicts of interest

The authors declare that they have no known competing financial interests or personal relationships that could have appeared to influence the work reported in this paper.

## Data Availability

The data supporting the findings of this study are available from the corresponding author upon reasonable request. All relevant experimental details and characterization results are included in the manuscript. No proprietary or confidential data were used.
